# Unlocking Tumor Aggressiveness in Endometrial Cancer: AI-Driven PET/CT Radiomics and Machine Learning for Prediction of High-Risk Tumor Histology

**DOI:** 10.3390/cancers18060905

**Published:** 2026-03-11

**Authors:** Samet Yagci, Evrim Erdemoglu, Mehmet Erdogan, Mustafa Avci, Ahmet Tunc, Ismail Ozkoc, Sevim Sureyya Sengul

**Affiliations:** 1 Department of Nuclear Medicine, Isparta City Hospital, Isparta 32000, Türkiye; 2 Department of Gynecologic Oncology, Faculty of Medicine, Süleyman Demirel University, Isparta 32260, Türkiye; evrimmd@yahoo.com; 3 Department of Nuclear Medicine, Faculty of Medicine, Süleyman Demirel University, Isparta 32260, Türkiye; mdr_erdogan@yahoo.com (M.E.); mustafaavci4864@gmail.com (M.A.); ahmettunc828@gmail.com (A.T.); sevim.sureyya.sengul@hotmail.com (S.S.S.); 4 Department of Nuclear Medicine, Antalya City Hospital, Antalya 07010, Türkiye; ozkocismail@gmail.com

**Keywords:** endometrial cancer, [18F]-FDG PET/CT, machine learning, radiomics

## Abstract

**Purpose:** Accurate preoperative risk stratification in endometrial cancer (EC) is essential for guiding surgical and therapeutic decisions. This study aimed to evaluate the discriminative performance of [18F]-FDG PET/CT-derived radiomic features combined with machine learning models for differentiating low-risk (LRH-EC) and high-risk histology (HRH-EC) subtypes. **Methods:** A total of 159 patients with histopathologically confirmed EC who underwent preoperative [18F]-FDG PET/CT were retrospectively analyzed. Radiomic features were extracted using LIFEx version 7.4.0 software following IBSI guidelines. After FDR correction and Pearson correlation–based redundancy reduction (|r| > 0.80), 16 radiomic features were retained for modeling. Three feature configurations (Conventional PET parameters, Radiomics16, and Combined) were evaluated. Machine learning models were developed using stratified 5-fold cross-validation. Model performance was assessed using AUC, accuracy, sensitivity, specificity, F1-score, Wilson confidence intervals, DeLong’s test, and McNemar’s test. **Results:** Artificial Neural Network (ANN) (AUC = 0.709) and Random Forest (RF) (AUC = 0.686) achieved the highest discriminative performance within the Radiomics16 feature set. No statistically significant superiority between algorithms or feature configurations was observed by DeLong analysis. However, McNemar’s test demonstrated significant patient-level classification differences for the Combined ANN model (*p* < 0.001). NGTDM_Coarseness and SUVmin emerged as the most influential features, reflecting tumor heterogeneity and metabolic activity. **Conclusions:** [18F]-FDG PET/CT-based radiomics combined with machine learning provides moderate yet consistent discrimination between LRH-EC and HRH-EC. While external validation is required, this approach may support noninvasive preoperative risk stratification in endometrial cancer.

## Simple Summary

Our work integrates advanced imaging analytics and machine learning to enhance preoperative risk stratification in endometrial cancer, an urgent requirement for personalized surgical planning. Our research explores the prognostic utility of [18F]-FDG PET/CT-derived metabolic, volumetric, and radiomic features in risk stratification of high-risk versus low-risk histological subtypes of endometrial cancer. This manuscript provides: Insights into novel PET radiomic biomarkers for endometrial cancer stratification. Evaluation of the clinical applicability of radiomics combined with machine learning in preoperative EC assessment. A step forward in improving non-invasive preoperative assessments.

## 1. Introduction

Endometrial cancer (EC) is the most common gynecological malignancy in developed countries worldwide. It is also recognized as the most common gynecological malignancy in the United States of America (USA), and the incidence of EC in USA is significantly higher than that of other developed countries. In the USA, estimated new cases in 2024 are 67,880 and estimated deaths in 2024 are 13.250 (SEER Database) [1]. Current practice standards assign endometrioid endometrial cancer (EEC) a FIGO (International Federation of Obstetrics and Gynaecology) grade according to the extent of glandular differentiation. FIGO grading should not be employed when the distinction of endometrioid or mucinous types is uncertain or cannot be determined [2]. All other endometrial tumor types feature an inherent tumor grade and p53 mutation; specifically, serous, clear cell, undifferentiated carcinomas, and carcinosarcomas are classified as high grade [3]. In practical practice, ascertaining FIGO grade is not always unequivocal [2,3].

Prognostication and risk stratification of EC is based on surgical staging reflecting the anatomical extent of disease. In the new revised FIGO 2023 staging system, non-anatomical parameters in stage I and II cancers such as specifically histological type and grade, lymphovascular invasion, and molecular group are introduced [4]. Aggressive histological subtypes, including serous, clear cell, undifferentiated, mixed, mesonephric-like, gastrointestinal mucinous type carcinoma, or carcinosarcomas with myometrial invasion, are now categorized as IIC disease according to the 2023 FIGO classification [5]. Low-grade (grade 1–2) EEC with limited myometrial invasion are categorized into lower-risk stage I substages, whereas high-grade (grade 3) tumors are assigned to higher-risk substages even with similar anatomical extent of disease. Furthermore, tumors with aggressive histology or high-grade features with myometrial invasion are upstaged to stage IIC. Thus, histological grade is no longer only a prognostic marker but also a staging determinant that may result in stage migration and has direct implications for surgical planning and adjuvant treatment decisions [5]. Preoperative risk stratification and correct identification of these parameters are important to triage the patient to surgery and tailor the surgery and hold utmost significance in vulnerable groups such as fertility desiring women or patients who are not ideal candidates for surgery. Schwameis et al. studied the stage migrations from FIGO 2009 staging to FIGO 2023 staging and their impact on survival [6]; In early-stage disease (stages I/II), 90% of stage transitions involved upshifts to a higher FIGO (sub)stage. The primary factors contributing to these upshifts were found to be aggressive histological subtypes or the presence of p53 alterations in carcinomas exhibiting myometrial invasion [6].

[18F]-FDG PET/CT has been verified to identify distant metastasis involving lymph node metastasis with high specificity and positive predictive value in GOG0233 trial [7], and NCCN 2025 version 1 guidelines [8] advise utilization of PET-CT, MRI, or CT in preoperative setting and for follow-up patients when metastasis is suspected. FDG, a glucose analogue, is the primary tracer utilized for the clinical evaluation of patients with gynecologic malignancies [9,10]. The fundamental justification for utilizing FDG in cancer is the Warburg effect, indicating an increase in glycolysis under aerobic conditions, which serves as a marker for malignancy [9]. The quantitative assessment of FDG intake predominantly utilizes the maximum standardized uptake value (SUVmax) due to its measurement simplicity; however, the standardized uptake value mean and peak, along with metabolic tumor volume (MTV) and total lesion glycolysis (TLG), are gaining wider acceptance [11]. Consequently, increased tumor aggressiveness, decreases disease free and overall survival has been previously associated with elevated SUVmax, MTV or TLG [9,12,13,14]. However, there is controversy on the role of these functional markers for predicting clinical and pathological markers [13].

Radiomics covers a range of computational methods that extract several quantitative variables characterizing image phenotypes, such as pixel intensity, shape, and texture, thereby yielding valuable metabolic and biological insights [15]. Radiomics features are divided into classes as first-order statistics, second-order statistics and high-order statistics. First-order features describe intensity distributions independent of spatial relationships, while second-order features evaluate relationships between adjacent voxels, and high-order features assess patterns across three or more voxels. Obtaining these quantitative parameters allows for objective and reproducible disease evaluation, patient classification, and the development of personalized treatment strategies [16,17,18]. In recent years, radiomics has emerged as a promising approach for tumor staging, early diagnosis, differentiation of histological subtypes, prognosis prediction, and treatment evaluation, providing insights beyond what is visible to the human eye. These features can reflect tumor heterogeneity and biology, supporting non-invasive, quantitative, and personalized clinical decision-making [19]. Integration of radiomics into clinical practice has the potential to improve patient outcomes by guiding treatment planning and risk stratification.

In particular, histological grade, one of the most important determinants of prognosis and staging in the revised FIGO 2023 system, has become a primary target of radiomic research. Meta-analyses of MRI-based radiomics have reported high sensitivity and specificity values in predicting high-grade tumors, suggesting that radiomics can reliably predict tumor aggressiveness preoperatively [20]. Beyond conventional clinicopathological factors, recent studies techniques have also shown that radiomics combined with machine learning or deep learning may predict molecular subtypes of EC, further enhancing personalized risk assessment [21].

However, PET/CT-based radiomic analysis for the prediction of aggressive histological subtypes in EC remains limited. To our knowledge, no previous study has comprehensively evaluated pretreatment [18F]-FDG PET/CT–derived metabolic, volumetric, and radiomic texture features using a machine learning framework for predicting aggressive histological subtypes in EC. Therefore, this study aimed to assess the effectiveness of preoperative [18F]-FDG PET/CT–derived metabolic and radiomic features, in combination with machine learning algorithms, for differentiating aggressive histological subtypes. We hypothesized that PET/CT-based metabolic and radiomic features integrated with machine learning methods could accurately identify aggressive histological subtypes in EC prior to surgery.

## 2. Materials and Methods

### 2.1. Patients

**Figure A1:**
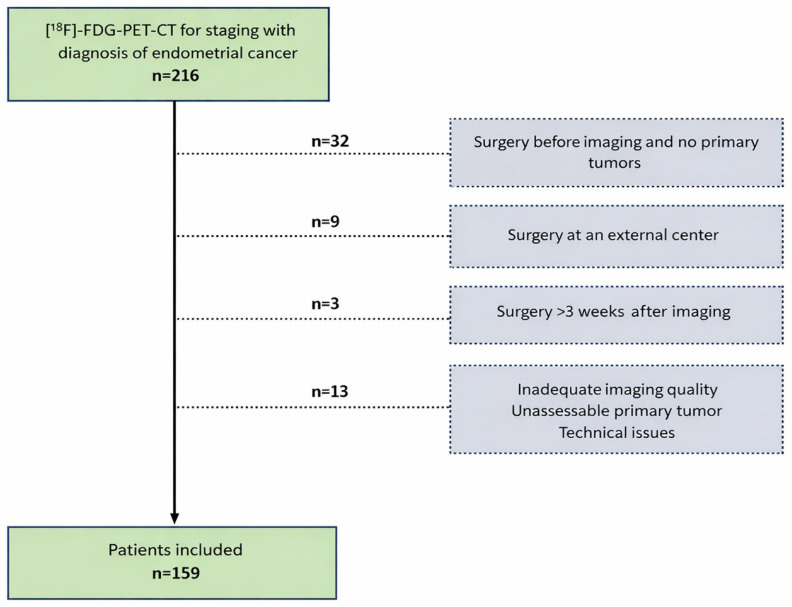
Criteria for Exclusion of Patients. The imaging data of 216 patients who [18F]-FDG PET/CT for staging with a diagnosis of EC were retrospectively reviewed. Thirty-two patients who had undergone surgery prior to imaging and had no primary tumor, nine patients who were operated on at an external center after imaging, three patients who underwent surgery more than three weeks after imaging, and thirteen patients whose imaging quality was inadequate for evaluation, whose primary tumor could not be clearly assessed, or whose imaging files could not be uploaded to the software due to technical issues were excluded from the study. After retrospective analysis of the imaging data, a total of 159 patients were included in the study.

The imaging data of 216 patients who underwent [18F]-FDG PET/CT for staging with a diagnosis of EC between November 2012 and October 2023 were retrospectively reviewed. Patients were included if they had histopathologically confirmed EC, underwent preoperative [18F]-FDG PET/CT at our institution, and subsequently received surgical treatment with available final histopathological results. Criteria for exclusion of patients in the study are given in Figure A1. After retrospective analysis of the imaging data, a total of 159 patients were included in the study.

The age at diagnosis, height, weight at the time of imaging, and pathology reports of the 159 included patients were retrieved from patient files and the hospital information system. Body mass index (BMI) was calculated using the recorded height and weight data. Pathology reports were reviewed to determine the subtypes and grades of the primary tumor. All patients underwent [18F]-FDG PET/CT within three weeks before surgery.

All patients had hysterectomy, with or without lymph node staging. Patients having a hysterectomy specimen exhibiting grade 1 or grade 2 endometrioid histology were classified as LRH-EC. Patients with serous cancer, carcinosarcoma, clear cell carcinoma, and undifferentiated carcinoma were categorized as HRH-EC. Since molecular classification could not be performed in our hospital, patients with grade 3 endometrioid histology were considered HRH-EC.

### 2.2. PET/CT Protocol

Informed consent was obtained from all patients before imaging. Foley catheterization and intravenous diuretics were administered to patients to reduce urinary activity in the bladder during imaging. All patients were instructed to drink 1.5 L of drinking water mixed with oral contrast agent (Kopaq 350 mg/mL) before the scan. After a fasting period of at least 4–6 h, blood glucose levels were measured using a glucometer. If the blood glucose level was <150 mg/dL, intravenous access was established using a 24-gauge cannula, and 3.7 MBq/kg (0.1 mCi/kg) of F [18F]-FDG was injected intravenously.

One hour after injection, low-dose CT was performed in the supine position for attenuation correction, scanning the entire body. CT parameters included a 64-slice multidetector system, 100–200 mAs, 120 kVp, slice thickness of 3 mm, rotation time of 0.5 s, table speed of 39 mm/s, and matrix size of 512 × 512. PET imaging was performed immediately afterward from the vertex to the mid-thigh in the supine position, with 1.5 min of acquisition time per bed position. The PET/CT scanner used was a Philips Gemini TF (Philips Medical Systems, Cleveland, OH, USA), operating in 3D mode with a resolution of 4.7 mm. The detector included LYSO (lutetium oxyorthosilicate) crystals with dimensions of 4 × 4 × 22 mm, totaling 28,336 crystals. The detector had pixelated architecture, a transverse field of view (FOV) of 576 mm, and an axial FOV of 180 mm. Acquired images were reconstructed using the row action maximum likelihood algorithm (RAMLA) and then evaluated.

### 2.3. Texture Analysis Protocol

[18F]-FDG PET/CT images in DICOM (Digital Imaging and Communications in Medicine) format were obtained for all patients. The images were imported into LIFEx software version 7.4.0 (www.lifexsoft.org), which complies with IBSI (Image Biomarker Standardisation Initiative) guidelines. The primary tumor was segmented in 3D using 40% of SUVmax as the threshold, a commonly used approach in PET radiomics to delineate metabolically active tumor volume and ensure reproducibility [22,23,24,25]. The segmentation was visually reviewed and verified by expert readers. After resampling the voxel size to 4 × 4 × 4 mm, textural matrices were calculated with absolute scale limits without SUV clipping, using a fixed number of 64 divisions (as suggested by IBSI). Voxel resampling to an isotropic grid was performed to standardize spatial resolution across patients and improve radiomic feature reproducibility, in accordance with IBSI-based radiomics guidelines and prior PET radiomics studies [20,26]. Quantitative parameters recorded for each lesion included SUVmax, SUVmean, SUVmin, MTV, TLG, shape features, histogram features, and 123 texture features derived from GLCM, NGTDM, GLRLM, and GLZSM matrices.

### 2.4. Statistical Analysis and Feature Selection

All statistical analyses were performed using R (version 4.5.2). Differences between the high-risk histology (HRH-EC) and low-risk histology (LRH-EC) groups were assessed using the two-sided Mann–Whitney U test and Welch’s *t*-test, as appropriate. To account for multiple comparisons among radiomic features, *p*-values were adjusted using the Benjamini–Hochberg FDR procedure. Radiomic features with FDR-adjusted Mann–Whitney U *p*-values < 0.05 were considered statistically significant and retained for further dimensionality reduction. To mitigate multicollinearity and reduce redundancy among significant radiomic variables, a Pearson correlation matrix was constructed, and highly correlated features (|r| > 0.80) were removed using the caret::findCorrelation function. This process resulted in a final set of 16 non-redundant radiomic features, which were subsequently used as input variables for machine learning analysis. Because feature selection and correlation filtering were performed prior to cross-validation, performance estimates should be interpreted as internally cross-validated rather than fully nested. Continuous variables are reported as median (Q1–Q3).

### 2.5. Feature Configurations

Three feature configurations were evaluated: (1) Conventional PET parameters, including SUVmax, SUVmean, SUVmin, metabolic tumor volume (MTV), and total lesion glycolysis (TLG); (2) Radiomics16 feature set identified after FDR-controlled screening followed by Pearson correlation–based redundancy reduction (|r| > 0.80); (3) Combined model integrating Conventional and Radiomics16 features.

### 2.6. Model Development and Validation

Machine learning models were trained separately for each feature configuration using stratified 5-fold cross-validation to preserve class distribution across folds. In each iteration, models were trained on four folds and evaluated on the remaining fold. Predicted class labels and predicted probabilities for held-out observations were stored and pooled across folds to compute overall performance metrics. The positive class was defined as HRH-EC. Algorithms evaluated (implemented using the R caret framework) included C5.0 decision tree, RF, SVM, ANN, Naive Bayes, and classification and regression tree (rpart). For algorithms requiring feature scaling (SVM and ANN), predictors were centered and scaled within the cross-validation workflow to prevent data leakage between training and validation folds. Hyperparameter tuning was performed within the same cross-validation framework to avoid information leakage. For RF, the mtry parameter was optimized; for SVM, the cost and sigma parameters were tuned; for ANN, size and decay were optimized; and for C5.0, the number of boosting trials was selected based on cross-validated ROC performance.

### 2.7. Performance Metrics

Confusion matrices were derived from pooled cross-validated predictions across all folds. From these matrices, the following performance metrics were calculated: Accuracy; Sensitivity; Specificity; Balanced accuracy; Cohen’s kappa; and F1-score (for the HRH-EC class). The pooled area under the receiver operating characteristic curve (AUC) was computed using ROC analysis based on pooled out-of-fold predicted probabilities for the HRH-EC class, with LRH-EC defined as the negative class. To provide more robust interval estimates for binomial performance measures (accuracy, sensitivity, specificity, positive predictive value, and negative predictive value), 95% confidence intervals were calculated using the Wilson score method.

### 2.8. Model Comparison Strategy

Model comparisons were conducted at two complementary levels. First, within each feature configuration, pairwise comparisons of AUC values between machine learning algorithms were performed using DeLong’s test for correlated ROC curves, applied to pooled out-of-fold predicted probabilities. *p*-values were adjusted using the Benjamini–Hochberg FDR procedure within each feature configuration. Second, within selected algorithms (RF and ANN), the impact of feature composition (Conventional vs. Radiomics vs. Combined) was evaluated using DeLong’s test. In addition to discrimination-based comparisons, classification differences between models were assessed using McNemar’s test based on paired cross-validated predictions. All statistical tests were two-sided, and *p*-values < 0.05 were considered statistically significant.

### 2.9. Feature Importance Analysis

Feature importance analysis was performed for the RF and ANN models trained on the Radiomics16 feature set. For RF, permutation-based importance was calculated within the caret framework and scaled relative to the most influential feature. For the ANN, importance was estimated using the Olden algorithm (NeuralNetTools package), and features were ranked according to the absolute magnitude of their importance scores. Importance values were interpreted within each model separately and were not directly compared across algorithms.

## 3. Results

A total of 159 patients were included in the study. All patients had hysterectomy and 108 of them were staged by complete pelvic lymphadenectomy (68%) with or without paraaortic lymphadenectomy. One hundred and eight patients were diagnosed with grade 1 or 2 endometroid cancer (LRH-EC). The high-risk EC group comprised 24 cases of serous cancer (47.1%), 5 cases of carcinosarcoma (9.8%), 4 cases of clear cell carcinoma (7.8%), 1 case of undifferentiated cancer (2%), and 17 cases of grade 3 EEC (33.3%), totaling 51 patients. Patient information is given in Table 1. Patients with HRH-EC (66 years old r: 37–85) were older than those with LRH-EC (57 years old r: 27–87, *p* < 0.001). The BMI of the HRH-EC group (30.7 kg/m^2^ r: 18.4–49.9) was significantly lower than that of the LRH-EC group (35.3 kg/m^2^ r: 18.9–49.9, *p* = 0.002).

A total of 66 radiomic parameters demonstrated statistically significant differences between the LRH-EC and HRH-EC groups after FDR correction (Mann–Whitney U test, Benjamini–Hochberg procedure) and are presented in Supplementary Table S1. Among these FDR-significant features, redundancy reduction using Pearson correlation filtering (|r| > 0.80) resulted in a final set of 16 non-redundant radiomic features retained for machine learning model development (Table 2). The resulting Radiomics16 feature set comprised texture-based parameters derived from GLCM, GLRLM, GLSZM, and NGTDM matrices, together with intensity-derived and morphological descriptors such as intensity variability metrics and surface-to-volume ratio. Collectively, this combination reflects a multidimensional PET-derived tumor characterization rather than a texture-only model.

Machine learning models trained using the 16 FDR-selected radiomic features demonstrated moderate discriminative performance (Table 3). Among the evaluated algorithms, the ANN achieved the highest AUC (0.709), followed closely by RF (0.686). The RF model demonstrated the highest overall accuracy (0.686) and specificity (0.806), whereas the ANN showed comparatively higher sensitivity for HRH classification (0.392). In contrast, the SVM exhibited markedly low sensitivity (0.059) despite high specificity (0.954), indicating a strong bias toward the majority (LRH) class. Naïve Bayes achieved the highest sensitivity (0.588) but at the expense of reduced specificity (0.750), reflecting a trade-off between false negatives and false positives. Overall, while several algorithms achieved comparable AUC values, differences in sensitivity–specificity balance were observed across models.

Among the evaluated classifiers, the ANN achieved the highest AUC, followed closely by RF. Although pairwise DeLong comparisons did not reveal statistically significant differences between algorithms (Supplementary Table S2), ANN and RF demonstrated the most favorable overall performance profiles in terms of discriminative ability and balanced sensitivity–specificity trade-off.

**Figure A2:**
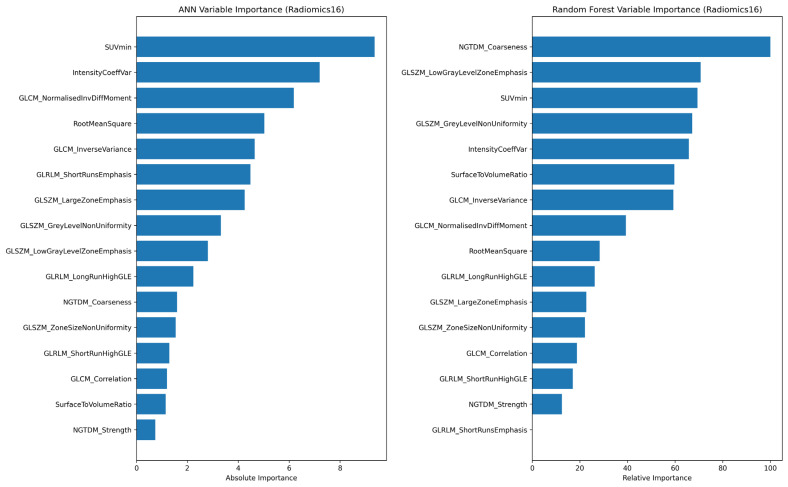
Variable importance analysis for the Radiomics16 feature set in Artificial Neural Network (ANN) and Random Forest (RF) models. The left panel shows absolute variable importance values derived from the ANN model, while the right panel presents relative importance scores from the RF model (scaled to 100 for the most influential feature). SUVmin emerged as the most influential predictor in the ANN model, whereas NGTDM_Coarseness demonstrated the highest importance in the RF model.

Feature importance analysis was performed for the RF and ANN models trained using the 16 radiomic features (Figure A2). In the RF, NGTDM_Coarseness emerged as the most influential variable, followed by GLSZM_LowGrayLevelZoneEmphasis, SUVmin, and GLSZM_GreyLevelNonUniformity. For the ANN model, feature importance was estimated using the Olden algorithm, with features ranked according to the absolute magnitude of their importance scores. Similar to the RF Model, NGTDM_Coarseness demonstrated substantial importance within the ANN framework. In addition, intensity-related parameters—particularly SUVmin and Intensity-Based Coefficient of Variation—showed strong contributions to model discrimination. Importance values were interpreted within each model independently due to methodological differences between permutation-based and weight-based estimation approaches.

Table 4 summarizes the cross-validated performance of the RF and ANN models across the Conventional, Radiomics, and Combined feature sets. For the RF model, the Radiomics feature set yielded the highest discriminative performance (AUC = 0.686), followed closely by the Combined model (AUC = 0.682), whereas the Conventional model demonstrated lower performance (AUC = 0.637). Sensitivity for HRH classification was modest across all RF models (0.33–0.43), while specificity remained relatively high (0.81–0.82). For the ANN model, the Radiomics feature set again achieved the highest AUC (0.709), followed by the Conventional model (AUC = 0.700) and the Combined model (AUC = 0.647). The Combined ANN model demonstrated the highest sensitivity for HRH detection (0.63), although this was accompanied by reduced specificity (0.71). Overall, Radiomics-based models provided comparable or slightly improved discriminative performance compared to Conventional features, whereas the addition of features in the Combined model did not result in a consistent improvement in AUC.

Table 5 presents pairwise comparisons between feature sets for the RF and ANN models using DeLong’s test for AUC comparison and McNemar’s test for paired classification differences.

For the RF, no statistically significant differences in AUC were observed between the Conventional, Radiomics, and Combined feature sets (all DeLong *p* > 0.05). Similarly, McNemar’s test did not demonstrate significant differences in classification outcomes across feature sets (all *p* > 0.05), indicating comparable predictive behavior between RF-based models.

For the ANN model, AUC comparisons between feature sets were also not statistically significant (all DeLong *p* > 0.05). However, McNemar’s test revealed significant classification differences when comparing the Combined model with both the Conventional (*p* = 3.0 × 10^−6^) and Radiomics (*p* = 3.1 × 10^−5^) models. This finding suggests that although overall discriminative performance (AUC) was similar, the Combined ANN model altered individual patient-level classifications relative to the other feature sets.

Overall, while no significant AUC superiority was demonstrated between feature sets, classification-level differences were observed in the ANN framework, particularly when the Combined feature set was used.

## 4. Discussion

This study demonstrates that [18F]-FDG PET/CT-derived radiomic features contain discriminative information for differentiating low-risk (LRH-EC) and high-risk (HRH-EC) endometrial cancer subtypes. After FDR-controlled screening, 16 radiomic features were retained and used for machine learning modeling. Among the evaluated algorithms, ANN and RF models achieved the highest discriminative performance, although no statistically significant superiority between algorithms was demonstrated by DeLong analysis. These findings indicate that radiomics-based models provide moderate but consistent discriminatory capability in preoperative risk stratification. In contrast, Naive Bayes and single decision tree–based models demonstrated comparatively lower sensitivity and overall discriminative performance. This may be related to their stronger independence assumptions and limited capacity to model complex, non-linear interactions among radiomic features. Radiomic descriptors often exhibit inter-feature dependencies and non-linear relationships, which may be better captured by ensemble or neural network–based approaches. Furthermore, the relatively modest sample size and class imbalance may have affected the stability of simpler models [27].

When feature sets were compared within the RF and ANN frameworks, no statistically significant differences in AUC were observed between Conventional, Radiomics, and Combined models according to DeLong’s test. This suggests that adding radiomic features to conventional PET parameters did not produce a statistically measurable improvement in global discriminative performance.

However, McNemar’s analysis revealed that the Combined ANN model produced significantly different patient-level classifications compared to both the Conventional and Radiomics-only ANN models. Although overall AUC values were comparable, this finding indicates that integrating radiomic and conventional parameters altered classification behavior at the individual level. Such differences may have clinical implications, as patient-level reclassification can influence preoperative risk assessment and management decisions even in the absence of large AUC changes.

Importantly, the Radiomics16 feature set incorporated not only texture features but also intensity-derived and volumetric parameters, including MTV and TLG. Therefore, the developed models reflect a comprehensive metabolic–morphological tumor profile rather than a texture-only approach. Taken together, these findings suggest that while conventional PET parameters retain diagnostic value, radiomics-driven machine learning models may offer a more integrative representation of tumor heterogeneity and aggressiveness.

Among texture-derived features, NGTDM_Coarseness emerged as the most influential parameter in the RF model. Higher coarseness values were observed in LRH-EC patients, indicating greater spatial uniformity, whereas HRH-EC tumors demonstrated lower coarseness, consistent with increased metabolic irregularity and intratumoral heterogeneity. Nakajo et al. reported that lower NGTDM_Coarseness values were significantly associated with disease progression and poorer survival outcomes in endometrial cancer, identifying coarseness as an independent prognostic parameter [28]. Similarly, Dang et al. demonstrated that lesions with higher coarseness values were less invasive, while lower values were associated with increased aggressiveness [29]. Coada et al. also reported a direct relationship between coarseness and progression-free survival [30].

**Figure A3:**
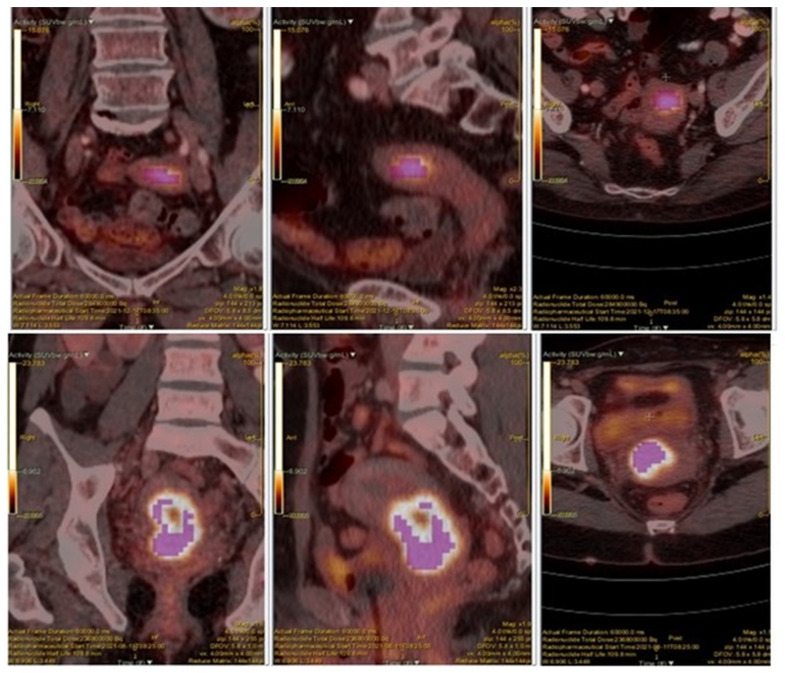
Representative [18F]-FDG PET/CT images illustrating differences in NGTDM_Coarseness between histological subtypes. The upper panel demonstrates a Grade 2 EEC with a higher coarseness value (0.05998), corresponding to a relatively homogeneous metabolic texture pattern. The lower panel shows a clear cell endometrial carcinoma with a markedly lower coarseness value (0.01207), reflecting increased intratumoral metabolic heterogeneity. These examples visually support the quantitative findings indicating lower coarseness values in high-risk histologies.

Although discrepancies in the direction of association have been described in certain tumor types, these differences likely reflect variations in segmentation thresholds, voxel resolution, and radiomics software platforms. Collectively, the consistent prominence of NGTDM_Coarseness in our machine learning models and its documented prognostic relevance in the literature support its potential role as a reproducible imaging biomarker reflecting tumor aggressiveness in endometrial cancer (Figure A3).

In addition to texture-derived features, SUVmin emerged as one of the most influential parameters in both the RF and ANN models. SUVmin values were significantly higher in HRH-EC compared to LRH-EC in our cohort. As SUVmin reflects the lowest metabolic activity within the segmented tumor volume, elevated values in HRH-EC may indicate relatively increased FDG uptake even in the least active tumor regions, potentially corresponding to a more aggressive metabolic phenotype. A similar observation was reported by Corica et al., who found higher SUVmin values in malignant solitary pulmonary nodules compared to benign lesions [31]. Collectively, these findings suggest that SUVmin may capture clinically relevant aspects of tumor biology beyond peak uptake metrics. The consistent importance of SUVmin across both machine learning algorithms indicates that conventional metabolic descriptors retain substantial predictive value. Importantly, this does not diminish the contribution of radiomics; rather, it highlights that tumor aggressiveness likely reflects the combined effect of global metabolic upregulation and spatial heterogeneity.

The application of radiomics and machine learning in endometrial cancer (EC) extends beyond PET/CT, encompassing studies based on ultrasound, CT, MRI, and hysteroscopic imaging for risk stratification, prediction of myometrial invasion, lymph node involvement, and distant metastases, as well as for guiding emerging preoperative and postoperative treatment strategies [32,33,34,35]. These approaches aim to enhance individualized decision-making and reduce unnecessary invasive interventions through more accurate, image-based characterization of tumor behavior. Among available modalities, PET/CT-based radiomics may offer distinct advantages by integrating metabolic and morphological information. Quantitative PET parameters such as SUVmax, TLG, and texture features can capture functional aspects of tumor biology that may not be fully reflected by purely morphological imaging techniques. Furthermore, the future incorporation of novel radiopharmaceuticals may further expand the biological insight obtainable from PET-based radiomics.

The present study also benefits from a rigorous methodological framework. All imaging data were acquired using a single PET/CT system with standardized acquisition and reconstruction protocols, minimizing technical variability. Histopathological confirmation following surgical staging was available for all cases, ensuring robust reference classification. A structured analytical pipeline was implemented, including FDR-controlled feature screening, stratified cross-validation, and complementary statistical comparisons using DeLong and McNemar tests. This approach allowed evaluation of both global discrimination performance and patient-level classification behavior, providing a comprehensive assessment of model performance. Furthermore, inclusion of lesions comprising at least 64 voxels ensured reliable extraction of higher-order texture features derived from GLCM, NGTDM, GLRLM, and GLSZM matrices, thereby enhancing radiomic stability and interpretability.

This study has several limitations. First, its retrospective single-center design may limit the generalizability of the findings. Although feature selection procedures were performed prior to cross-validation and stratified 5-fold cross-validation was implemented to reduce the risk of overfitting, the sample size remains relatively modest in relation to the number of extracted radiomic features, which may affect model stability. In addition, class imbalance within the dataset may have contributed to the relatively low sensitivity observed in some machine learning algorithms.

Another important limitation is the absence of complete molecular classification for all tumors. According to the FIGO 2023 system [5], molecular subtyping plays a central role in risk stratification, particularly in high-grade EEC. However, due to the retrospective design and the unavailability of molecular profiling in a subset of patients, grade 3 endometrioid tumors were categorized as high-risk histology in accordance with the FIGO 2023 framework. This approach may have introduced potential misclassification bias and could have influenced model performance.

Additional limitations relate to tumor segmentation and reproducibility, which remain fundamental challenges in radiomics research. Although the segmentation threshold was selected based on commonly reported values and ensured consistent delineation, it may not fully capture the entire tumor volume, particularly in the presence of background activity. A formal interobserver or intraobserver variability analysis was not performed, although all segmentations were independently reviewed by experienced nuclear medicine physicians without clinically relevant discrepancies. Furthermore, while radiomic features were extracted using widely accepted software, validation across different platforms is required to confirm reproducibility.

Finally, radiomic features may reflect not only intrinsic tumor heterogeneity but also image noise and technical factors, and given the spatial resolution constraints of PET/CT imaging, they may not fully represent cellular-level heterogeneity. External validation in independent multicenter cohorts is therefore necessary before clinical implementation can be considered. Nevertheless, even moderate predictive performance may hold clinical relevance in the preoperative setting, where noninvasive tools capable of identifying aggressive histological subtypes could complement existing risk stratification and surgical planning strategies.

## 5. Conclusions

In conclusion, [18F]-FDG PET/CT-derived radiomic features combined with machine learning models provide moderate yet consistent discriminative capability for differentiating low-risk and high-risk endometrial cancer subtypes. Although no statistically significant superiority was observed between algorithms or feature configurations in terms of AUC, integration of radiomic and conventional PET parameters influenced patient-level classification, highlighting the potential added value of multidimensional imaging analysis. Among the evaluated features, NGTDM_Coarseness and SUVmin emerged as prominent contributors, suggesting that both spatial heterogeneity and global metabolic activity play complementary roles in reflecting tumor aggressiveness. While further multicenter validation and methodological standardization are required, PET-based radiomics may serve as a noninvasive tool to support preoperative risk stratification and individualized clinical decision-making in endometrial cancer.

## Figures and Tables

**Table 1:** Pathological Type and Grade Information.

	Type		Total (%)
	LRH-EC (%)	HRH-EC (%)	
Pathological Type	108 (67,9)	51 (32,1)	159
Endometrioid	108 (100)	17 (33,3)	125 (78,6)
Carcinosarcoma	0 (0)	5 (9,8)	5 (3,1)
Serous	0 (0)	24 (47,1)	24 (15,1)
Clear Cell	0 (0)	4 (7,8)	4 (2,5)
Undifferentiated	0 (0)	1 (2)	1 (0,6)
GRADE			
Non-endometrioid	0 (0)	34 (66,7)	34 (21,4)
Grade 1	64 (59,3)	0 (0)	64 (40,3)
Grade 2	44 (40,7)	0 (0)	44 (27,7)
Grade 3	0 (0)	17 (33,3)	17 (10,7)

**Table 2:** Radiomics16 feature set obtained after FDR correction and Pearson correlation-based redundancy reduction.

Feature	LRH-EC Median (Q1–Q3)	HRH-EC Median (Q1–Q3)	FDR-Adjusted *p*-Value
NGTDM_Coarseness	0.031 (0.020–0.044)	0.017 (0.008–0.028)	**<0.001**
GLCM_InverseVariance	0.000273 (0.000051–0.000715)	0.000031 (0.000003–0.000295)	**0.001**
GLSZM_GreyLevelNonUniformity	4.25 (3.27–6.67)	7.47 (4.02–14.00)	**0.001**
IntensityBasedCoefficientOfVariation	0.258 (0.243–0.283)	0.239 (0.231–0.255)	**0.002**
SurfaceToVolumeRatio (mm^−1^)	0.304 (0.256–0.341)	0.246 (0.208–0.304)	**0.002**
Histogram_RootMeanSquare	0.022 (0.015–0.028)	0.014 (0.009–0.020)	**0.005**
SUVmin	3.72 (2.45–4.72)	4.36 (3.20–6.27)	**0.015**
GLCM_Correlation	0.341 (0.262–0.422)	0.412 (0.330–0.489)	**0.015**
GLSZM_LowGrayLevelZoneEmphasis	0.00234 (0.00125–0.00545)	0.00136 (0.00091–0.00312)	**0.020**
GLRLM_LongRunHighGreyLevelEmphasis	656 (327–1193)	1050 (495–1729)	**0.020**
GLCM_NormalisedInverseDifferenceMoment	0.945 (0.930–0.958)	0.954 (0.937–0.967)	**0.027**
GLSZM_LargeZoneEmphasis	4.72 (3.22–11.6)	7.32 (4.26–29.8)	**0.028**
GLRLM_ShortRunHighGreyLevelEmphasis	533 (247–1040)	914 (416–1504)	**0.032**
GLSZM_ZoneSizeNonUniformity	37.3 (23.4–73.0)	63.9 (28.2–138)	**0.035**
GLRLM_ShortRunsEmphasis	0.969 (0.954–0.977)	0.964 (0.939–0.972)	**0.037**
NGTDM_Strength	9.15 (4.78–15.4)	5.93 (2.71–11.3)	**0.037**

**Table 3:** Machine learning performance with 95% Wilson confidence intervals (5-fold CV, Radiomics16).

Model	Accuracy (95% CI)	Sensitivity (95% CI)	Specificity (95% CI)	PPV (95% CI)	NPV (95% CI)	F1-Score (95% CI)
C5.0	0.711 (0.636–0.776)	0.412 (0.288–0.548)	0.852 (0.773–0.907)	0.568 (0.409–0.713)	0.754 (0.671–0.822)	0.477 (0.376–0.580)
Artificial Neural Network (nnet)	0.711 (0.636–0.776)	0.510 (0.377–0.641)	0.806 (0.721–0.869)	0.553 (0.412–0.686)	0.777 (0.691–0.844)	0.531 (0.433–0.626)
Random Forest	0.730 (0.656–0.793)	0.431 (0.305–0.567)	0.870 (0.794–0.921)	0.611 (0.449–0.752)	0.764 (0.682–0.831)	0.506 (0.403–0.608)
Support Vector Machine	0.667 (0.590–0.735)	0.059 (0.020–0.159)	0.954 (0.896–0.980)	0.375 (0.137–0.694)	0.682 (0.604–0.751)	0.102 (0.047–0.205)
Naive Bayes	0.698 (0.623–0.764)	0.588 (0.452–0.712)	0.750 (0.661–0.822)	0.526 (0.399–0.650)	0.794 (0.706–0.861)	0.556 (0.462–0.646)
Decision Tree (rpart)	0.660 (0.584–0.729)	0.431 (0.305–0.567)	0.769 (0.681–0.838)	0.468 (0.333–0.608)	0.741 (0.653–0.813)	0.449 (0.354–0.548)

Point estimates were derived from pooled out-of-fold predictions obtained using stratified 5-fold cross-validation. Confidence intervals for binomial proportions were calculated using the Wilson score method. HRH-EC was defined as the positive class.

**Table 4:** Performance of RF and ANN Across Feature Sets (Pooled 5-fold CV).

Algorithm	Feature Set	Accuracy	Sensitivity	Specificity	Balanced Accuracy	Kappa	F1 (HRH-EC)	AUC
RF	Conventional	0.6541	0.3333	0.8056	0.5694	0.1489	0.3820	0.6373
RF	Radiomics	0.6855	0.4314	0.8056	0.6185	0.2472	0.4681	0.6855
RF	Combined	0.6918	0.4314	0.8148	0.6231	0.2582	0.4731	0.6823
ANN	Conventional	0.7296	0.3725	0.8981	0.6353	0.3037	0.4691	0.7003
ANN	Radiomics	0.7044	0.3922	0.8519	0.6220	0.2645	0.4598	0.7086
ANN	Combined	0.6855	0.6275	0.7130	0.6702	0.3205	0.5614	0.6471

Values were computed from pooled out-of-fold predictions obtained using stratified 5-fold cross-validation. HRH was defined as the positive class.

**Table 5:** Feature Set Comparisons Within RF and ANN (DeLong and McNemar Tests).

Algorithm	Comparison	AUC 1	AUC 2	DeLong *p*-Value	McNemar *p*-Value
RF	Conventional vs. Radiomics	0.6373	0.6855	0.092	0.383
RF	Conventional vs. Combined	0.6373	0.6823	0.067	0.540
RF	Radiomics vs. Combined	0.6855	0.6823	0.709	1.000
ANN	Conventional vs. Radiomics	0.7003	0.7086	0.780	0.211
ANN	Conventional vs. Combined	0.7003	0.6471	0.221	**<0.001**
ANN	Radiomics vs. Combined	0.7086	0.6471	0.096	**<0.001**

AUC differences were assessed using DeLong’s test for correlated ROC curves based on pooled out-of-fold predicted probabilities. Classification differences were evaluated using McNemar’s test. Very small *p*-values are reported as *p* < 0.001.

## Data Availability

The datasets generated during and/or analysed during the current study are available from the corresponding author on reasonable request.

## Supplementary Materials

The following supporting information can be downloaded at: https://www.mdpi.com/article/10.3390/cancers18060905/s1; Supplementary Table S1: Radiomic features significantly associated with histological risk group following FDR-adjusted Mann–Whitney U analysis; Supplementary Table S2: Pairwise AUC Comparisons Between Machine Learning Algorithms (Radiomics16 Feature Set).

## Abbreviations

The following abbreviations are used in this manuscript:

FDG	Fluorodeoxyglucose
PET	Positron Emission Tomography
CT	Computed Tomography
HRH-EC	High Risk Histological Subtypes of Endometrial Cancer
LRH-EC	Low Risk Histological Subtypes of Endometrial Cancer
FDR	False Discovery Rate
SVM	Support Vector Machine
SUV	Standardized Uptake Value
AUC	Area Under Curve
USA	United States of America
FIGO	International Federation of Obstetrics and Gynaecology
MTV	Metabolic Tumor Volume
TLG	Total Lesion Glycolysis
ROI	Region of Interest
BMI	Body Mass Index
DICOM	Digital Imaging and Communications in Medicine
IBSI	Image Biomarker Standardisation Initiative
PPV	Positive Predictive Value
NPV	Negative Predictive Value
OS	Overall Survival
PCA	Principal Component Analysis
EEC	Endometrioid Endometrial Cancer
ANN	Artificial Neural Network
RF	Random Forest
