# Supplementary material for: Unlocking Tumor Aggressiveness in Endometrial Cancer: AI-Driven PET/CT Radiomics and Machine Learning for Prediction of High-Risk Tumor Histology
Source: Cancers (Basel). 2026 Mar 11;18(6):905. doi: 10.3390/cancers18060905 (PMC13025313; doi:10.3390/cancers18060905)
Supplement: Supplementary file 1 [file cancers-18-00905-s001.zip › Supplementary Table S1..pdf]

Supplementary Table S1. Radiomic features significantly associated with histological risk group following FDR-adjusted Mann–Whitney U analysis.

Feature	LRH_median_IQR	HRH_median_IQR	MWU_p	FDR_MWU
**SUVmin**	3.718 (2.445–4.724)	4.357 (3.202–6.270)	0.003	0.015
**SUVmean**	7.048 (4.583–9.756)	9.156 (6.133–11.773)	0.010	0.028
**SUVmax**	12.089 (8.915–16.924)	15.557 (10.262–20.362)	0.014	0.030
**MTV**	8.896 (5.680–15.936)	18.048 (9.280–39.648)	<0.001	<0.001
**TLG**	60.904 (34.093–124.898)	175.166 (88.551–358.748)	<0.001	<0.001
**Morphological Volume**	8405.333 (5308.667–15184.000)	17970.667 (8729.333–39672.000)	<0.001	<0.001
**Integrated Intensity**	56660.660 (31777.039–119449.465)	171352.361 (84444.490–346946.834)	<0.001	<0.001
**Voxel Count**	139.000 (88.750–249.000)	291.000 (145.000–635.000)	<0.001	<0.001
**Sphere Diameter**	25.708 (22.137–31.221)	32.887 (26.071–42.654)	<0.001	<0.001
**Surface Area**	2544.290 (1821.973–4005.717)	4179.752 (2766.917–9145.318)	<0.001	<0.001
**Surface To Volume Ratio**	0.304 (0.256–0.341)	0.246 (0.208–0.304)	<0.001	0.002
**Maximum3D Diameter**	40.200 (33.226–52.268)	50.279 (38.261–72.765)	<0.001	0.006
**Energy**	7221.404 (2864.577–22477.348)	23794.073 (10769.269–65066.891)	<0.001	<0.001
**Intensıty-Based Coefficient Of Variation**	0.258 (0.243–0.283)	0.239 (0.231–0.255)	<0.001	0.002
**Intensıty-Based Quartile Coefficient Of Dispersion**	0.198 (0.177–0.214)	0.182 (0.156–0.200)	0.006	0.020
**Intensıty-Based Area Under Curve CIVH**	2.364 (1.591–3.203)	3.028 (2.062–3.832)	0.011	0.028
**Intensıty-Based Root Mean**	7.223 (4.893–10.073)	9.415 (6.331–12.165)	0.012	0.029

**Square**				
**Intensıty-Based 10thIntensity Percentile**	4.954 (3.095–6.933)	6.775 (3.877–8.469)	0.004	0.019
**Intensıty-Based 25thIntensity Percentile**	5.635 (3.795–7.986)	7.617 (4.768–9.469)	0.006	0.020
**Intensıty-Based 50thIntensity Percentile**	6.756 (4.438–9.695)	8.862 (5.839–11.965)	0.009	0.026
**Intensıty-Based 75thIntensity Percentile**	8.247 (5.605–11.595)	10.348 (7.188–14.182)	0.021	0.038
**Intensity-Histogram Coefficient Of Variation**	0.252 (0.235–0.276)	0.230 (0.216–0.246)	<0.001	<0.001
**Intensity-Histogram Quartile Coefficient Of Dispersion**	0.193 (0.176–0.212)	0.175 (0.157–0.200)	0.004	0.018
**Intensity-Histogram Root Mean Square**	0.022 (0.015–0.028)	0.014 (0.009–0.020)	<0.001	0.005
**Intensity-Histogram Mode**	19.000 (12.750–27.000)	24.000 (16.500–35.500)	0.011	0.028
**Intensity-Histogram Mean**	23.044 (15.169–31.701)	29.800 (20.138–38.194)	0.011	0.028
**Intensity-Histogram Area Under Curve CIVH**	7.201 (4.740–9.907)	9.313 (6.293–11.936)	0.011	0.028
**Intensity-Histogram Maximum Grey Level**	39.000 (29.000–54.500)	50.000 (33.500–64.000)	0.025	0.046
**Intensity-Histogram Minimum Grey Level**	12.000 (8.000–16.000)	14.000 (10.500–20.500)	0.003	0.016
**Intensity-Histogram 10th Percentile**	16.000 (10.750–23.000)	22.000 (13.000–27.500)	0.005	0.020
**Intensity-Histogram**	19.000 (13.000–26.000)	25.000 (15.500–31.000)	0.005	0.020

**25th Percentile**				
**Intensity-Histogram 50th Percentile**	22.000 (14.750–31.250)	29.000 (19.000–39.000)	0.009	0.026
**Intensity-Histogram 75th Percentile**	26.500 (18.750–38.000)	34.000 (23.500–46.000)	0.020	0.037
**Intensity-Histogram Minimum Histogram Gradient**	-4.500 (-7.125–-3.000)	-6.500 (-10.750–-4.000)	0.007	0.023
**Intensity-Histogram Minimum Histogram Gradient Grey Level**	21.000 (14.000–29.250)	29.000 (18.000–41.000)	0.008	0.024
**Intensity-Histogram Maximum Histogram Gradient**	7.500 (4.000–12.125)	14.000 (6.500–28.750)	<0.001	0.001
**Intensity-Histogram Maximum Histogram Gradient Grey Level**	15.500 (11.000–23.000)	21.000 (13.500–27.000)	0.010	0.027
**GLCM Inverse Variance**	0.00027 (0 – 0.05208)	0.00003 (0 – 0.00495)	<0.001	0.001
**GLCM Correlation**	0.341 (0.262–0.422)	0.412 (0.330–0.489)	0.003	0.015
**GLCM Normalised Inverse Difference**	0.845 (0.825–0.866)	0.862 (0.837–0.880)	0.004	0.017
**GLCM Normalised Inverse Difference Moment**	0.945 (0.930–0.958)	0.954 (0.937–0.967)	0.010	0.027
**GLCM Autocorrelation**	597.083 (280.910–1159.103)	1015.200 (477.650–1685.157)	0.013	0.030
**GLCM Joint Average**	24.269 (16.584–33.767)	31.568 (21.645–40.591)	0.014	0.030
**GLCM Sum Average**	48.539 (33.168–67.533)	63.135 (43.291–81.183)	0.014	0.030

**NGTDM Coarseness**	0.031 (0.020–0.044)	0.017 (0.008–0.028)	<0.001	<0.001
**NGTDM Strength**	9.146 (4.780–15.357)	5.928 (2.712–11.330)	0.020	0.037
**GLRLM Short Runs Emphasis**	0.969 (0.954–0.977)	0.964 (0.939–0.972)	0.019	0.037
**GLRLM Long Runs Emphasis**	1.127 (1.098–1.213)	1.155 (1.119–1.295)	0.014	0.030
**GLRLM Low Grey Level RunEmphasis**	0.002 (0.001–0.005)	0.001 (0.001–0.003)	0.006	0.020
**GLRLM High Grey Level Run Emphasis**	555.367 (260.346–1066.320)	937.093 (431.270–1547.794)	0.013	0.030
**GLRLM Short Run Low Grey Level Emphasis**	0.002 (0.001–0.005)	0.001 (0.001–0.003)	0.005	0.020
**GLRLM Long Run Low Grey Level Emphasis**	0.003 (0.001–0.006)	0.001 (0.001–0.004)	0.019	0.037
**GLRLM Short Run High Grey Level Emphasis**	533.014 (247.335–1039.715)	913.526 (415.888–1504.379)	0.016	0.032
**GLRLM Long Run High Grey Level Emphasis**	655.940 (326.849–1193.086)	1050.029 (495.446–1729.316)	0.006	0.020
**GLRLM Run Percentage**	0.960 (0.938–0.969)	0.953 (0.919–0.964)	0.013	0.030
**GLRLM Run Length Non Uniformity**	125.814 (81.363–205.641)	229.830 (126.644–494.127)	<0.001	0.001
**GLRLM Grey Level Non Uniformity**	8.750 (5.448–16.249)	16.402 (7.955–37.837)	<0.001	0.002
**GLSZM Large Zone Emphasis**	4.716 (3.221–11.609)	7.321 (4.265–29.757)	0.011	0.028
**GLSZM Small Zone Low Grey Level Emphasis**	0.002 (0.001–0.003)	0.001 (0.001–0.002)	0.001	0.007
**GLSZM Large Zone High Grey Level Emphasis**	3193.067 (1888.770–6810.290)	8160.941 (3961.720–20874.048)	<0.001	<0.001
**GLSZM Low Gray Level Zone Emphasis**	0.002 (0.001–0.005)	0.001 (0.001–0.003)	0.005	0.020
**GLSZM High Gray Level Zone Emphasis**	554.671 (269.739–1011.651)	935.779 (421.439–1481.579)	0.014	0.030
**GLSZM Grey**	4.249 (3.274–	7.471 (4.016–	<0.001	0.001

**Level NonUniformity**	6.671)	14.006)		
**GLSZM Zone Size Entropy**	5.166 (4.850–5.555)	5.557 (5.121–5.849)	0.004	0.017
**GLSZM Zone Size Variance**	1.943 (0.913–7.109)	3.871 (1.538–18.655)	0.007	0.023
**GLSZM Zone Size NonUniformity**	37.286 (23.403–73.040)	63.854 (28.178–138.177)	0.018	0.035

Features are ordered as follows: Conventional PET parameters (SUVmin, SUVmean, SUVmax, MTV, TLG), followed by Morphological, Intensity-based, Intensity-histogram, GLCM, NGTDM, GLRLM, and GLSZM features. Continuous variables are presented as median (Q1–Q3). P-values were obtained using the Mann–Whitney U test and adjusted using the Benjamini–Hochberg FDR procedure.
