# Supplementary material for: Unlocking Tumor Aggressiveness in Endometrial Cancer: AI-Driven PET/CT Radiomics and Machine Learning for Prediction of High-Risk Tumor Histology
Source: Cancers (Basel). 2026 Mar 11;18(6):905. doi: 10.3390/cancers18060905 (PMC13025313; doi:10.3390/cancers18060905)
Supplement: Supplementary file 1 [file cancers-18-00905-s001.zip › Supplementary Table S2..pdf]

Supplementary Table S2. Pairwise AUC Comparisons Between Machine Learning Algorithms (Radiomics16 Feature Set)

Algorithm 1	Algorithm 2	AUC 1	AUC 2	DeLong p-value	FDR-adjusted p-value
ANN	DT	0.718	0.631	0.183	0.642
SVM	ANN	0.643	0.718	0.234	0.642
RF	DT	0.712	0.631	0.241	0.642
RF	SVM	0.712	0.643	0.303	0.642
C50	DT	0.698	0.631	0.321	0.642

Pairwise comparisons of AUC values were performed using DeLong's test for correlated ROC curves. P-values were adjusted using the Benjamini–Hochberg false discovery rate (FDR) procedure. No statistically significant differences were observed between algorithms (all FDR-adjusted p-values > 0.05).
